# Transient Perivascular Inflammation of the Carotid Artery (TIPIC) Syndrome: An Atypical Cause of Neck Pain

**DOI:** 10.7759/cureus.41275

**Published:** 2023-07-02

**Authors:** Jéssica A Abreu, Cláudia A Rocha, Sofia G Cruz, João S Barreira

**Affiliations:** 1 Internal Medicine, Hospital de Vila Franca de Xira, Vila Franca de Xira, PRT; 2 Serviço de Saúde da Região Autónoma da Madeira, Centro de Saúde de Machico, Funchal, PRT; 3 Cerebrovascular Disease Unit, Hospital Professor Doutor Fernando Fonseca, Amadora, PRT

**Keywords:** transient perivascular inflammation of the carotid artery, fay syndrome, inflammation, neck pain, carotidynia, tipic syndrome

## Abstract

Transient perivascular inflammation of the carotid artery (TIPIC) is an uncommon condition characterized by inflammation of the carotid artery wall, leading to unilateral neck pain. While TIPIC has been acknowledged by the International Classification of Headache Disorders, only a few patient series have been published thus far.

The clinical presentation of TIPIC syndrome typically manifests as unilateral neck pain localized specifically over the carotid artery. This pain is accompanied by ipsilateral tenderness and increased arterial pulsation. The condition commonly follows a self-limited course or demonstrates a favorable response to treatment with nonsteroidal anti-inflammatory drugs. When evaluating patients with suspected TIPIC syndrome, conducting a comprehensive assessment of their clinical history is imperative, while utilizing imaging studies to exclude any potential structural abnormalities of the carotid artery effectively.

The authors present a case involving a 57-year-old woman who presented with a two-month history of persistent left cervical pain and tenderness. Ultrasonography findings revealed indirect indications of inflammation in the intima-media layer of the carotid artery, suggestive of carotidynia. Notably, other significant differential diagnoses such as aneurysms or carotid dissection were ruled out. Over the course of the evaluation, there was a gradual and spontaneous improvement in both clinical symptoms and radiological findings, indicating the resolution of the inflammatory process as confirmed by imaging follow-up.

This case presents a rare and atypical manifestation of transient neck pain attributed to TIPIC.

## Introduction

Carotidynia was originally documented in 1927 by Temple Fay, a renowned American neurologist and neurosurgeon, who identified it as a condition featuring tenderness and pain localized at the carotid bifurcation [1]. Initially categorized as an idiopathic syndrome associated with neck pain in the inaugural edition of the International Classification of Headache Disorders (ICHD) in 1988 [2], the subsequent reporting of imaging irregularities necessitated the establishment of precise diagnostic criteria [3].

In 2004, the nomenclature "Carotidynia" was substituted with the acronym TIPIC, denoting transient perivascular inflammation of the carotid artery. TIPIC syndrome is distinguished by unilateral or bilateral cervical pain, tenderness localized over the common carotid bifurcation, and augmented pulse attributable to arterial wall inflammation. The pain may extend to the ipsilateral side of the face or ear, or cause headache.

The diagnostic criteria for TIPIC syndrome entail a consensus on the following parameters: acute pain specifically localized over the carotid artery, with or without radiation to the head; evidence of eccentric perivascular infiltration as on imaging; exclusion of alternative vascular or nonvascular diagnoses through imaging; and observable improvement within a span of 14 days, either spontaneously or in response to anti-inflammatory treatment [4,5].

Although the exact prevalence of TIPIC syndrome remains unknown, current evidence indicates that it may be a relatively uncommon clinical condition, with reported incidences of up to 2.8% in small-scale studies involving patients presenting with acute neck pain [6].

The diagnosis of TIPIC syndrome is established through a comprehensive assessment involving clinical examination and imaging studies. These investigations reveal evidence of vascular and perivascular inflammation, characterized by circumferential thickening of the carotid wall at the distal common carotid or carotid bifurcation area. Importantly, luminal narrowing is typically absent, and Doppler ultrasound results commonly indicate normal cardiovascular hemodynamics in the carotid artery, indicating preserved cardiovascular hemodynamics of the carotid artery. It is noteworthy that the distinct features of vessel wall inflammation observed in TIPIC syndrome, such as the absence of calcification and a limited clinical course, differentiate it from atherosclerotic plaque [5].

Further investigation is imperative to exclude alternative differential diagnoses, including carotid artery dissection, aneurysm, hematoma, or thrombosis. It is also important to consider conditions such as giant cell arteritis, cervical abscess, cervical lymphadenitis, thyroiditis, and trigeminal neuralgia [4].

TIPIC syndrome follows a benign and self-limited trajectory, typically resolving within two weeks, either spontaneously or with the aid of nonsteroidal anti-inflammatory drugs [4-8].

The likelihood of recurrence is minimized, but initial follow-up is still recommended [5].

## Case presentation

The authors present a clinical case involving a 57-year-old woman who had well-managed arterial hypertension and depression. The patient presented to the emergency department with a clinical history of moderate-intensity left neck pain localized specifically over the carotid artery. The pain was accompanied by local tenderness and worsened with movements of the head and neck. It had been persisting for a period of two months, with no preceding upper respiratory tract infection or history of trauma. The patient denied experiencing headaches, syncope, or neurological deficits. Initially, she sought consultation with her general practitioner who conducted a neck ultrasound examination. The results of the ultrasound indicated abnormalities, leading to a recommendation for immediate referral to the hospital emergency department. During the physical examination, the patient experienced increased pain upon superficial palpation. The oropharyngeal mucosa appeared normal, and no palpable neck mass or lymphadenopathy was detected.

**Figure 1:**
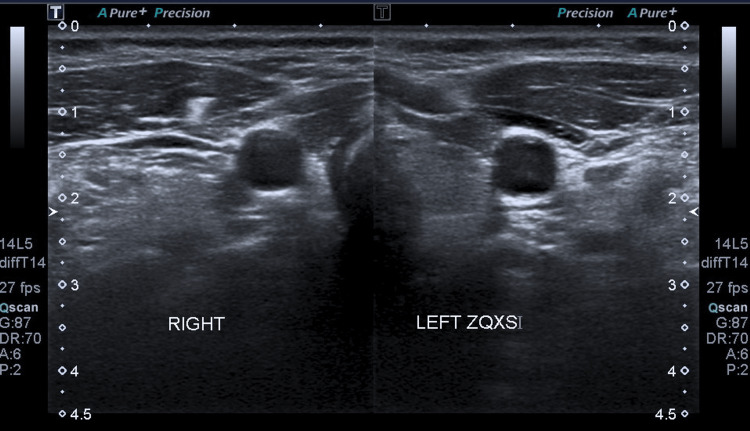
Neck ultrasonography showing perivascular hyperechogenicity on the left common carotid artery.

Blood samples were normal. Upon repeat neck ultrasonography, focal parietal enlargement of the common carotid artery and hyperechogenicity of the periaortic tissue were observed (Figure 1), indicating the presence of an inflammatory process.

**Figure 2:**
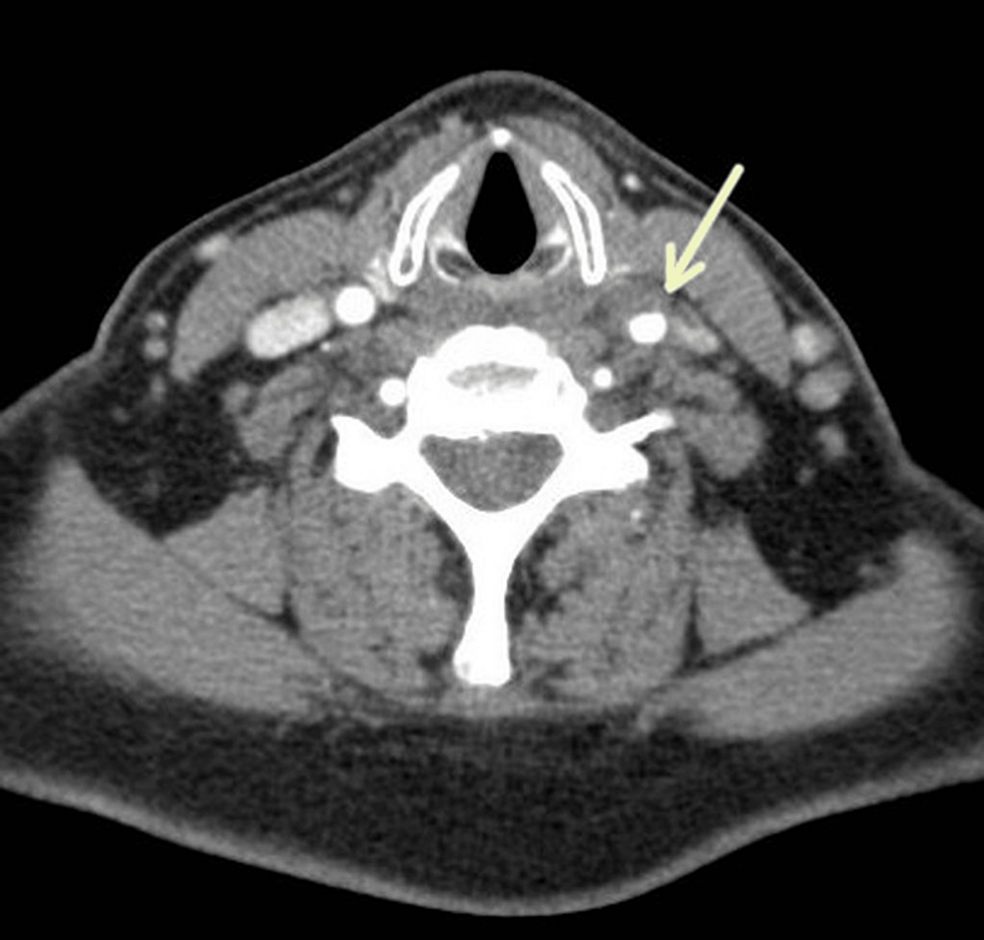
Axial contrast-enhanced CT image showing left common carotid artery reduced caliber (arrow).

Doppler evaluation showed an increased resistance index (RI) on both common carotid arteries (RI = 0.81 on the right and 0.76 on the left), with no hemodynamic significance. Contrast computed tomography (CT) (Figure 2) excluded vascular abnormalities, such as aortic dissection or aneurysm, but showed indirect signs of inflammation (left common carotid artery anterior wall reduced caliber and anterior wall flattening) without arterial blood flow turbulence or flow velocity acceleration. This was suggestive of TIPIC syndrome.

**Figure 3:**
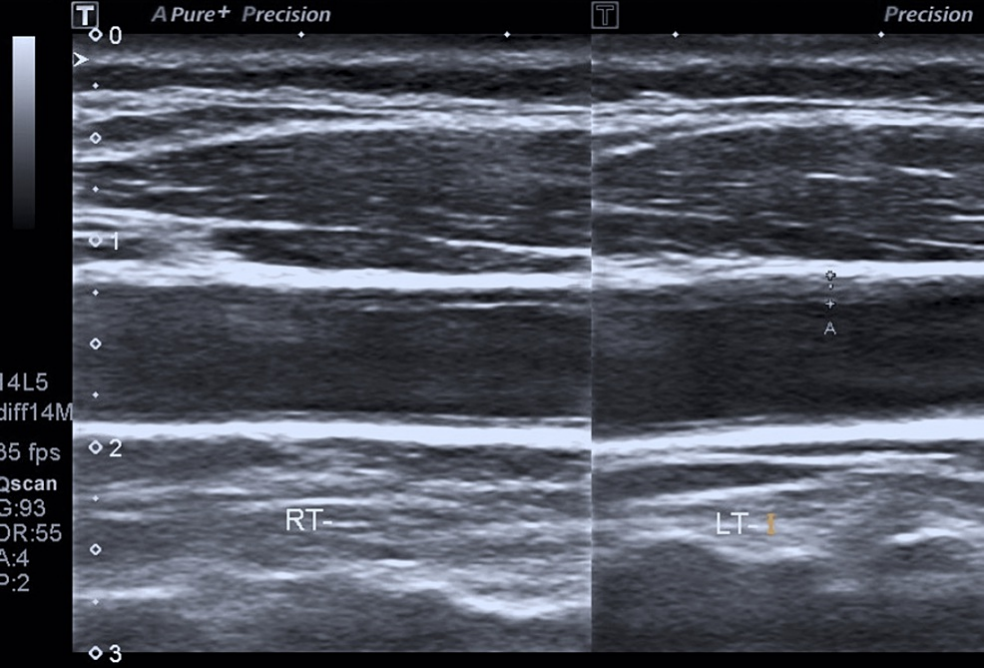
Neck ultrasonography revealing thickened vessel wall (1.4 mm) of the left common carotid artery.

A short course of nonsteroidal anti-inflammatory drugs was prescribed, and a follow-up review was conducted after two months. During the review, the patient reported relief from symptoms. Ultrasonography re-evaluation revealed normal echogenicity of previously affected perivascular tissues. However, there was still observed enlargement of the left carotid artery intima-media wall (1.4 mm bilateral; Figure 3), with no indications of vasculitis. Doppler measurement indicated a sustained increase in the resistance index (RI = 0.86 on the right and 0.80 on the left).

There was no recurrence within the 12-month follow-up.

## Discussion

We present a case of a woman diagnosed with TIPIC syndrome, in which the evolution was benign but prolonged over time.

Her symptoms included pain in the left neck over the carotid artery, accompanied by local tenderness. She described moderate-intensity pain that worsened with head and neck movements. The pain associated with TIPIC is typically described as dull and throbbing, ranging in severity from mild to severe. Swallowing, chewing, or any movement of the head or neck can also exacerbate the pain. There may be swelling or fullness over the carotid bifurcation, along with a more pronounced carotid pulse. Constitutional symptoms such as fever or malaise were absent [6].

In contrast to the typical clinical course of TIPIC syndrome, which is self-limited to two weeks with spontaneous resolution or response to anti-inflammatory drugs, this case had a longer duration of symptoms extending to two months, prompting further investigation.

Imaging studies revealed the presence of inflammatory processes within the common carotid artery, as evidenced by increased intima-media thickening and elevated resistance index. These findings are consistent with the diagnosis of TIPIC syndrome, as reported in previous studies [4,5,7]. Importantly, other potential vascular abnormalities necessitating surgical intervention, such as carotid artery dissection, aneurysm, or thrombosis, were thoroughly ruled out. Furthermore, comprehensive evaluations excluded non-vascular causes of cervical pain, including neck tumors, temporomandibular joint disorder, peritonsillar abscess, Eagle syndrome, and dental disease. It is crucial to approach the management of such cases on an individual basis, considering the unique characteristics and circumstances of each patient.

Histologic evaluation was not performed in this case, as the condition followed a benign course. However, previous literature provides insights into TIPIC through a limited number of reports involving vascular biopsies. These studies have uncovered various findings, including vascular and fibroblast proliferation, nonspecific inflammatory processes, and the absence of neoplasms, infections, granulomas, giant cells, or vasculitis [7].

## Conclusions

TIPIC syndrome is an infrequent and self-limiting condition characterized by benign neck pain, which typically resolves spontaneously. In this particular case, the clinical course appeared to deviate from the expected duration associated with this syndrome, albeit without prognostic implications. It is crucial to distinguish this inflammatory process from atherosclerotic disease and thoroughly exclude any local vessel wall abnormalities. Although rare, this entity should be considered within the differential diagnosis of unilateral neck pain, and an initial investigative assessment can be initiated by a general practitioner.
